# Domain-Domain Interactions Underlying Herpesvirus-Human Protein-Protein Interaction Networks

**DOI:** 10.1371/journal.pone.0021724

**Published:** 2011-07-07

**Authors:** Zohar Itzhaki

**Affiliations:** Department of Microbiology and Molecular Genetics, IMRIC, Faculty of Medicine, The Hebrew University of Jerusalem, Jerusalem, Israel; University of Oxford, United Kingdom

## Abstract

Protein-domains play an important role in mediating protein-protein interactions. Furthermore, the same domain-pairs mediate different interactions in different contexts and in various organisms, and therefore domain-pairs are considered as the building blocks of interactome networks. Here we extend these principles to the host-virus interface and find the domain-pairs that potentially mediate human-herpesvirus interactions. Notably, we find that the same domain-pairs used by other organisms for mediating their interactions underlie statistically significant fractions of human-virus protein inter-interaction networks. Our analysis shows that viral domains tend to interact with human domains that are hubs in the human domain-domain interaction network. This may enable the virus to easily interfere with a variety of mechanisms and processes involving various and different human proteins carrying the relevant hub domain. Comparative genomics analysis provides hints at a molecular mechanism by which the virus acquired some of its interacting domains from its human host.

## Introduction

Protein-protein interactions (PPIs) play a key role in the dialogue between viruses and their hosts. Datasets of host-virus protein interactions based on large- and small-scale studies are available for a growing number of viruses, particularly herpesviruses [1], [2], [3], [4], [5], [6], [7], [8]. These data expand our knowledge of viral proteins involved in such interactions and shed light on cellular processes interfered by the virus. Describing host-virus protein interactions as a network in which the nodes are the proteins and the edges are the interactions between them, enables their study by network analysis tools. Using such tools it was shown that the global properties of host-virus interaction networks resemble those of cellular networks. In particular, it was shown that there are human proteins that are hubs in this network (interacting with many viral partners), and also that viral proteins tend to interact with ‘bottlenecks’ (human proteins that are central to many paths in the cellular network [9].

It is well established that many of the PPIs are achieved through domain-domain interactions (DDIs, [10], [11], [12], [13]). In recent years, databases of interacting domain-pairs were published, based on data from protein complexes solved by crystallography (*e.g:* [14], [15]. Using these data, we and others [16], [17] showed that statistically significant fractions of the cellular networks of various organisms, ranging from *Escherichia coli* to human, can be attributed to the experimentally-determined interacting domain-pairs. Furthermore, we showed that different organisms use the same interacting domain-pairs, and suggested that there is a limited repertoire of domain-pairs that underlie interactome networks, and that DDIs can be considered as the building blocks of the interactome.

Here we take this approach one step further and examine whether conserved DDIs also mediate host-virus interaction networks (in particular human-herpesvirus interactions). We point to the domain-pairs that may mediate these interactions and provide a dataset of viral and human domains and domain-pairs involved in herpesvirus infection. We find that indeed, statistically significant fractions of the human-virus interactions can be attributed to the intra-species conserved DDIs. Analyzing this dataset enables us to further investigate evolutionary and molecular aspects of both the domains (human and virus domains) involved in the interactions, and also the human-virus domains-pairs. We show that viral domains tend to interact with human hub domains and find hints at a molecular mechanism by which the virus may have acquired interacting domains from its human host.

### Results and Discussion

This study focuses on three herpesviruses: Herpes Simplex Virus type I (HSV), Epstein-Barr Virus (EBV) and Kaposi Sarcoma-associated Human Virus (KSHV). For each virus we compiled a dataset of intra-viral and human-virus protein interactions, as detailed in Table 1, based on both small- and large-scale studies [1], [2], [4], [5], [6], [7], [8]. Each protein in the datasets was labeled by its domains according to Pfam database [18]. The dataset of interacting domain-pairs was based on two sources: (i) domain-pairs based on crystallographic data, derived from 3DID database [14], and (ii) domain-pairs derived from known single-domain interacting proteins from various organisms [3], [8]. The latter is based on the widely accepted notion that stable protein interactions are mediated by DDIs, and therefore, conceivably, single-domain proteins interact using their sole domains.

**Table 1:** DDI-PPI mapping of intra-viral and human-virus protein interactions.

Network type	Type	HSV	EBV	KSHV
Host-virus protein interactions	(a) Number of PPIs	88	442	15
	(b) Number of PPIs with known domains	71	331	9
	(c) Number of PPIs attributed to DDIs	39	240	4
	(d) Fraction of PPIs attributed to DDIs (c/b)	0.55	0.73	0.44
	(e) p-value	<0.001	<0.001	<0.001
	(f) Number of DDIs / viral domains	36 / 11	144 / 24	6 / 4
Intra- viral protein interactions	(a) Number of PPIs	76	71	155
	(b) Number of PPIs with known domains	68	48	106
	(c) Number of PPIs attributed to DDIs	51	43	89
	(d) Fraction of PPIs attributed to DDIs (c/b)	0.75	0.90	0.84
	(e) p-value	<0.001	<0.001	<0.001
	(f) Number of DDIs / viral domains	51 / 36	39 / 36	89 / 38

We next asked whether the human-virus PPIs could be attributed to the interacting domain-pairs. If two interacting proteins contained domains documented as interacting, where one domain resides in one protein and the other domain resides in the interacting partner, the interaction of these proteins was attributed to this/these domain-pair/s. For EBV we also used information on the protein interacting regions reported by Calderwood *et al.* [1], and mapped them to the domains (see Dataset S1). For each virus and each dataset (of intra-viral or human-virus PPI) we counted the number of PPIs with known domains (each of the interacting proteins consists of at least one known domain), and then counted the number of protein interactions that could be attributed to the interacting domain-pairs (Table 1). To evaluate the statistical significance of our findings, we generated 1,000 random interaction networks for each virus and network type (intra-virus, human-virus), preserving the number of nodes (number of different proteins in the network), the degree of each node, and whether the node represents a human or a viral protein. These networks were generated by randomly selecting viral and human proteins from the virus and human proteomes, respectively. For each random network we repeated the same analysis performed on the actual PPI networks: we counted the number of PPIs with known domains, and then counted the number of interactions that could be attributed to the interacting domain-pairs. We compared the latter number with that number in the actual networks. The fraction of random networks where this count was equal to or exceeded the count in the actual network provided the statistical significance (Table 1).

For each of the viruses included in the study we found a statistically significant fraction of both the intra-viral and human-virus interactions that could be attributed to the interacting domain-pairs (Table 1 and Figure S1). To avoid bias, when carrying out the analysis for a specific virus, we excluded the DDIs derived from that specific virus from the interacting domain-pair database. The results were still statistically significant. Of note, some intra-viral and human-virus PPIs were found to be mediated by the same domain-pairs that mediate PPIs in human. Hence, we concluded that there are conserved domain-pairs that not only mediate interactions within organisms but also in the host-virus interface.

Since human-virus interactions involve human domains that are also used for mediating interactions in the human cellular interaction network, it is possible that viral and human domains compete for interaction with the same human domains. For example, the Kaposi sarcoma's Cyclin homolog (Uniprot [19] accession Q98147) interacts with the human protein CDK6 (Cyclin-dependent kinase 6, Q00534), which is also known to interact with various human Cyclin proteins. The interaction between the viral Cyclin homolog and the human CDK6 may deregulate the host cell cycle progression, which is beneficial for the virus [20]. These two interactions could be attributed to the Cyclin N-terminal domain (Pfam accession PF00134) found in both human and virus, and Protein Kinase domain (PF00069) found in human.

**Figure 1:**
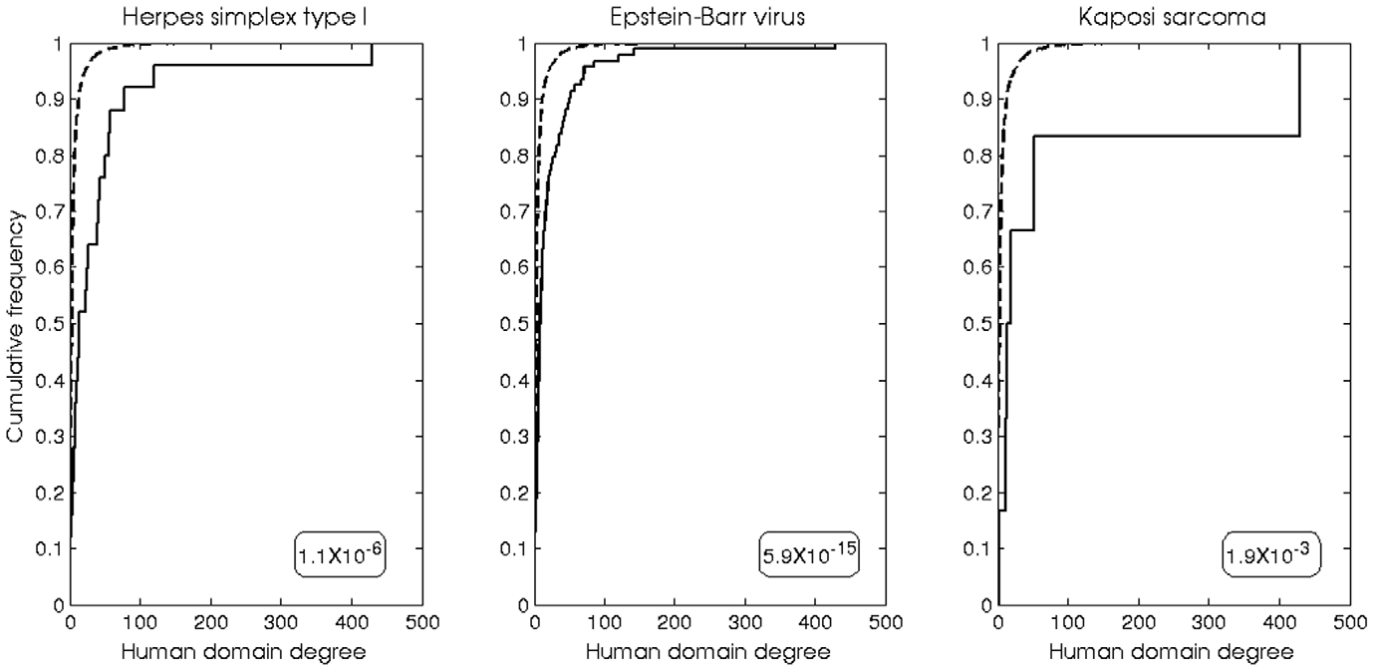
Herpesviruses domains tend to interact with human hub domains. Comparison between the degrees of human domains targeted by viral domains (“target”, solid lines) and the degrees of human domains that were not found to be targeted by viral domains (“non target”, dashed lines). The human domain degrees were calculated according to the human single-domain PPI network (see text). Human domains that are targeted by the viral domains have a higher degree than the human domains that are not targeted by the viral domains. These finding are statistically significant by a Kolomogorov Smirnof (p-values are given in rounded box at the right bottom of each graph).

Interestingly, many of the human domains to which the host-virus interactions were attributed, are domains involved in interactions with multiple domains in the human cellular network (hub-domains). To assess the statistical significance of this phenomenon we generated the human DDI network based on human single-domain protein complexes, where there is no doubt as to the domains mediating the interactions. In this network each node is a domain and edges connect between nodes of interacting domains. The degree of a node is the number of domains it interacts with. We compared the degrees of the viral-interacting human domains (based on the above analysis) to the degrees of all other human domains (not associated with viral domain interactions). We found that human domains involved in interactions with viral domains have statistically significantly higher degrees in comparison to human domains not involved in interactions with viral domains (Figure 1). These human domains are capable of interactions with a variety of other domains, and may mediate various interactions in the host's cellular network. Interactions between the viral domains and such human domains may be beneficial for the virus, and enable the virus to interfere with multiple mechanisms and processes involving various human proteins carrying the relevant domain.

**Figure 2:**
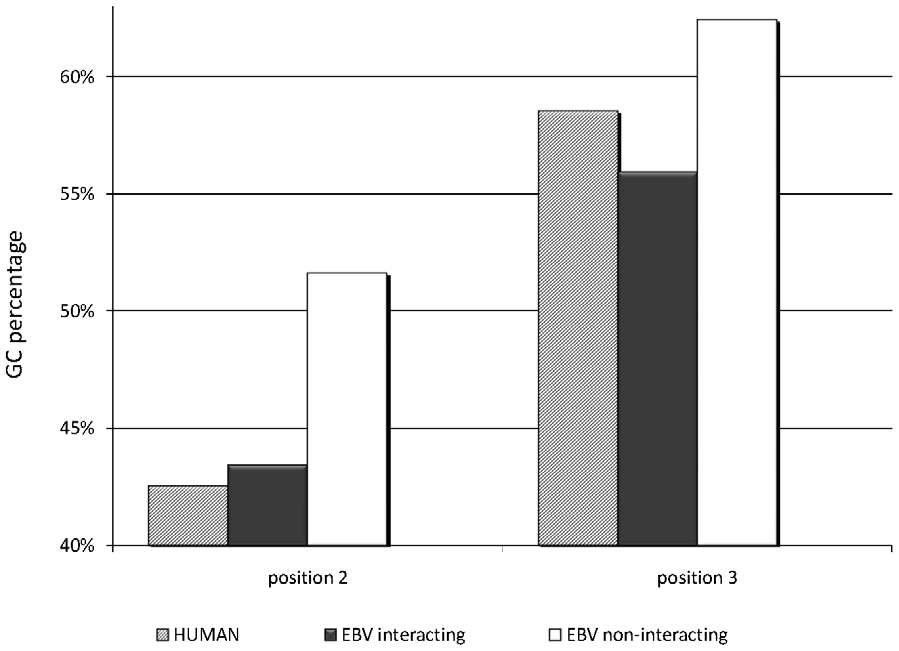
Comparison between interacting and non-interacting viral domains. Comparison of the average GC content in the 2nd and 3rd positions of the codon between (1) the human genome (gray bars), (2) viral domains that mediate host-virus protein interactions (black bars), and (3) viral domains were not found to mediate host-virus protein interactions (white bars). This comparison demonstrates the similarity in the GC content of the viral domains mediating the host-virus protein interactions and the human genome, as well as the differences in the GC content between the viral domains mediating the host-virus interactions and the viral domains not-mediating these interactions. A Pitman Permutation Test for the differences between the mediating and the non-mediating domains yielded statistically significant results (p-values: 2nd position: 2.3×10^−3^,3rd position: 0.01, calculated using StatXact version 9 software (Cytel Inc., Cambridge, MA)).

We have shown that human-virus protein interactions can be attributed to specific domain-pairs. As it is known that viruses can acquire proteins from their host, it is interesting to find out whether viral domains involved in interactions are more similar to human domains than other viral domains that were not found in the analysis as involved in interactions. It was recently reported that the general similarity in GC content between virus and host is indicative of viral adaptation towards the host [21], [22]. We applied this analysis to the data of EBV, in which 24 viral domains were included in the DDI repertoire. First, we characterized the interacting domains by the GC contents of the DNA sequences encoding them [21], [22]. We computed the GC content in the second and third positions of codons in regions encoding: 1) viral domains that interact with human domains. 2) viral domains that do not interact with human domains. 3) human genome using Kazusa's *codon usage database* [23]. We used a Pitman Permutation Test [24] and compared the GC content of the interacting and the non-interacting domains. We found that there are statistically significant differences between these two groups. Notably, the interacting domains were more similar to the human domains in their GC content (Figure 2). The significant similarity between the human genes and the viral domains interacting with the host may hint that the origin of interacting domains is in the human host. In order to further investigate this hypothesis we compared the DNA sequences encoding the interacting domains to the exons of all human genes and found that many of them were statistically significantly similar to human exons (Blast [25] E<1×10^−4^). Furthermore, for several interacting domains we found that their sub-domain sequence organization is consistent with that of the human gene. For example, the viral Interleukin-10 homolog protein (Uniprot accession P03180) interacts with the human protein Interleukin-10 receptor subunit alpha (IL10RA, Q13651) and consequently may control the inflammatory response, affect the antigen presentation by the cells and consequently may enable the virus to escape from the host immune detection [26], [27]. This interaction is mediated via the viral domain Interleukin 10 (Pfam accession PF00726). Pairwise alignment of the domain's DNA to the human mature mRNA of the gene interleukin-10 (IL-10) reveals statistically significant similarities and consistency in the order of the domain fragments. This finding further suggests that the virus gained domains capable of interaction with host proteins by acquiring host mRNA during the course of evolution. This conjecture is in accord with the well established knowledge of viruses, especially large DNA viruses, acquiring genes from their eukaryotic host [28], [29], and further demonstrates the sophistication in the evolution of viruses, empowering them an efficient interaction with their host.

## Supporting Information

Figure S1 **Distributions of fractions of PPIs attributed to DDIs in the random networks.** The fractions in the actual networks are written in the upper right box in each graph.
(TIF)

Dataset S1 **The domain-pairs that potentially mediate the human-virus and intra-virus PPIs.**
(XLS)
