# Supplementary figures and images for: Domain-Domain Interactions Underlying Herpesvirus-Human Protein-Protein Interaction Networks
Source: PLoS One. 2011 Jul 7;6(7):e21724. doi: 10.1371/journal.pone.0021724 (PMC3131297; doi:10.1371/journal.pone.0021724)

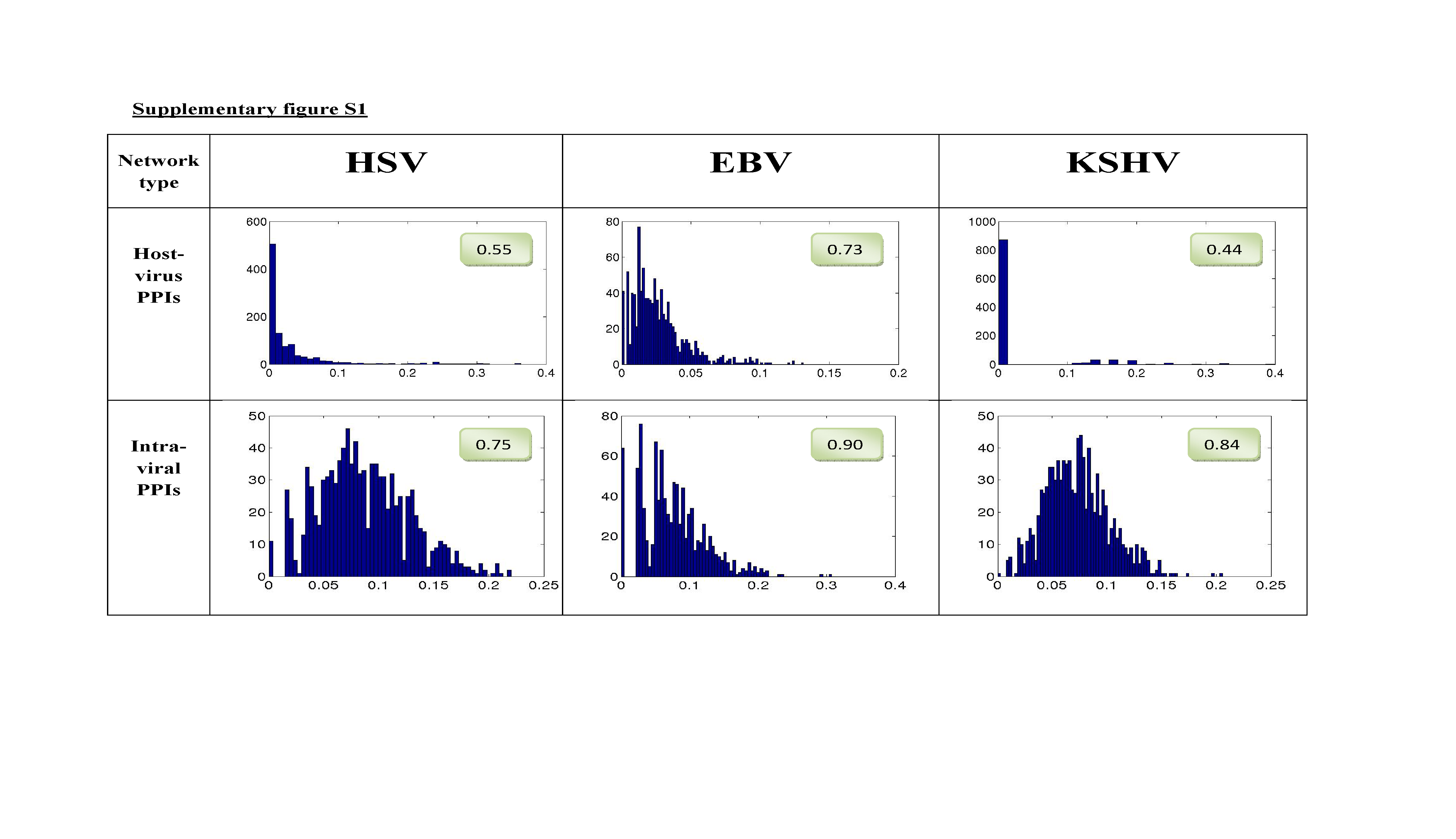

Supplement: Figure S1 — Distributions of fractions of PPIs attributed to DDIs in the random networks. The fractions in the actual networks are written in the upper right box in each graph. (TIF) [file pone.0021724.s001.tif]
